# Utility of multispectral imaging for nuclear classification of routine clinical histopathology imagery

**DOI:** 10.1186/1471-2121-8-S1-S8

**Published:** 2007-07-10

**Authors:** Laura E Boucheron, Zhiqiang Bi, Neal R Harvey, BS Manjunath, David L Rimm

**Affiliations:** 1 Electrical and Computer Engineering Department, University of California, Santa Barbara, CA, 93106, USA; 2 Space and Remote Sensing Sciences, Los Alamos National Laboratory, P.O. Box 1663, Los Alamos, NM, 87545, USA; 3 Department of Pathology, Yale University School of Medicine, P.O. Box 208023, New Haven, CT, 06520, USA

## Abstract

**Background:**

We present an analysis of the utility of multispectral versus standard RGB imagery for routine H&E stained histopathology images, in particular for pixel-level classification of nuclei. Our multispectral imagery has 29 spectral bands, spaced 10 nm within the visual range of 420–700 nm. It has been hypothesized that the additional spectral bands contain further information useful for classification as compared to the 3 standard bands of RGB imagery. We present analyses of our data designed to test this hypothesis.

**Results:**

For classification using all available image bands, we find the best performance (equal tradeoff between detection rate and false alarm rate) is obtained from either the multispectral or our "ccd" RGB imagery, with an overall increase in performance of 0.79% compared to the next best performing image type. For classification using single image bands, the single best multispectral band (in the red portion of the spectrum) gave a performance increase of 0.57%, compared to performance of the single best RGB band (red). Additionally, red bands had the highest coefficients/preference in our classifiers. Principal components analysis of the multispectral imagery indicates only two significant image bands, which is not surprising given the presence of two stains.

**Conclusion:**

Our results indicate that multispectral imagery for routine H&E stained histopathology provides minimal additional spectral information for a pixel-level nuclear classification task than would standard RGB imagery.

## Background

The use of multispectral imaging capabilities is relatively new to the field of cyto- and histo-pathology, particularly for transmitted brightfield microscopy [1,2]. Recent publications (e.g., [3-6]) have begun to explore the use of extra information contained in such spectral data (29–33 wavelengths in the visible spectrum, from 400 nm to 720 nm, spaced 10 nm apart), in particular for multiply stained (>2 stains) specimens. Specifically, there have been comparisons of spectral unmixing algorithms (to separate constituent dyes) which demonstrate the advantage of multispectral data [5,7]. The added benefit of multispectral imaging for analysis of routine H&E cyto/histopathology imagery, however, is still largely unknown, although some promising results are presented in [6].

While the use of multispectral light microscopy is new to cyto/histopathology, many researchers have used single or dual narrow-band filters to enhance imagery for particular stains, most using a red filter (or the red channel of an RGB image) for enhancement of Hematoxylin or Feulgen staining [8-12], and some using a green filter for enhancement of Feulgen staining [13-16].

We present analyses of our multispectral data designed to test the hypothesis that the additional spectral bands contain more information useful for classification as compared to the 3 standard bands of RGB microscopy imagery. The work presented here is an extension of the work presented in [17].

## Results and discussion

### Classification using all image bands

**Figure 1:**
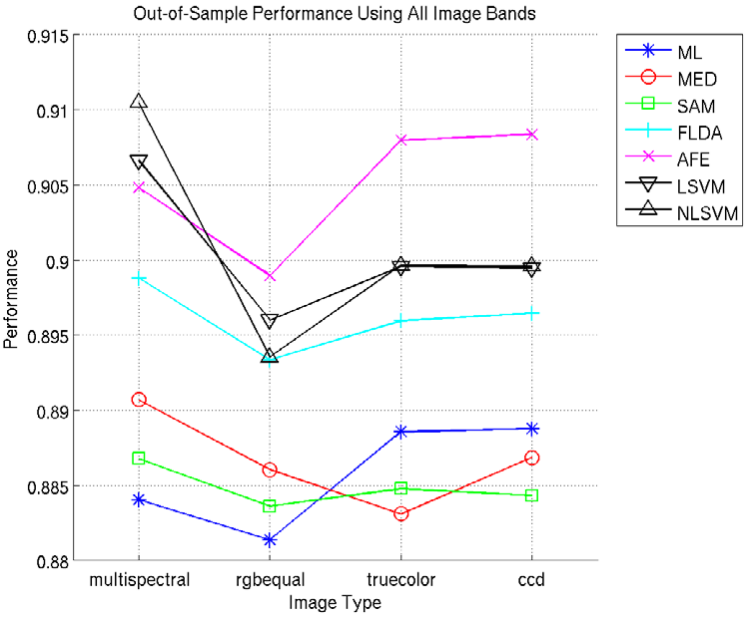
**Average performance using all available image bands**. Performance (Equation 1) is presented within the range [0, 1].

We split our dataset in (approximately) half to create a set of training images and a set of test images; half each of the benign and malignant subsets were randomly assigned to the training or test set to allow for even representation of benign and malignant characteristics in both sets. (One less benign image is included in the training set.) Applying all six classifiers to each image, using all available image bands, and averaging over the images contained in the test (out-of-sample) set, we achieve the results shown in Figure 1. Since the AFE tool GENIE is stochastic, we average ten independent runs. We would like to point out that the quadratic SVM (NLSVM) was run with only 10% of the total training data.

The best performance *P *is obtained with either the multispectral or ccd image stacks, with ML and AFE performing better with ccd imagery, and MED, FLDA, SAM, and both SVMs performing better with multispectral. It is important to note, however, that these increases in performance are only, on average, 0.79%. We use a paired Wilcoxon signed rank test to determine the statistical significance of these differences in performance, and show our results in Table 1; we see that less than half of these differences are statistically significant.

**Table 1:** Wilcoxon p-values for performances of multispectral versus RGB imagery.

**Classifier**	**Image**	**Image**		
		rgbequal	truecolor	ccd
**ML**	multi	0.5440	0.0978	0.0822
**MED**	multi	**0.0000**	**0.0001**	**0.0000**
**SAM**	multi	**0.0057**	0.7343	0.8290
**FLDA**	multi	0.0656	0.0752	0.1156
**AFE**	multi	**0.0030**	0.1109	**0.0285**
**LSVM**	multi	**0.0012**	0.6288	0.4284
**NLSVM**	multi	**0.0000**	**0.0047**	**0.0060**

Wilcoxon paired signed-rank test p-values are presented to 5 significant digits, and bold entries correspond to statistical significance at the p-value of 0.05.

We have shown in this section, using a pairwise Wilcoxon signed rank test, that only a few performance differences between multispectral and RGB imagery are statistically significant. Furthermore, we note that these statistically significant differences are 0.46%, 0.76%, and 0.38% increase in favor of multispectral imagery over rgbequal, truecolor, and ccd, respectively, for MED; 0.32% in favor of multispectral over rgbequal for SAM; 0.58% in favor of multispectral over rgbequal and 0.35% in favor of ccd over multispectral for AFE; 1.06% in favor of multispectral over rgbequal for LSVM; and 1.7%, 1.1%, and 1.1% in favor of multispectral over rgbequal, truecolor, and ccd, respectively, for NLSVM.

### Classification using single image bands

**Figure 2:**
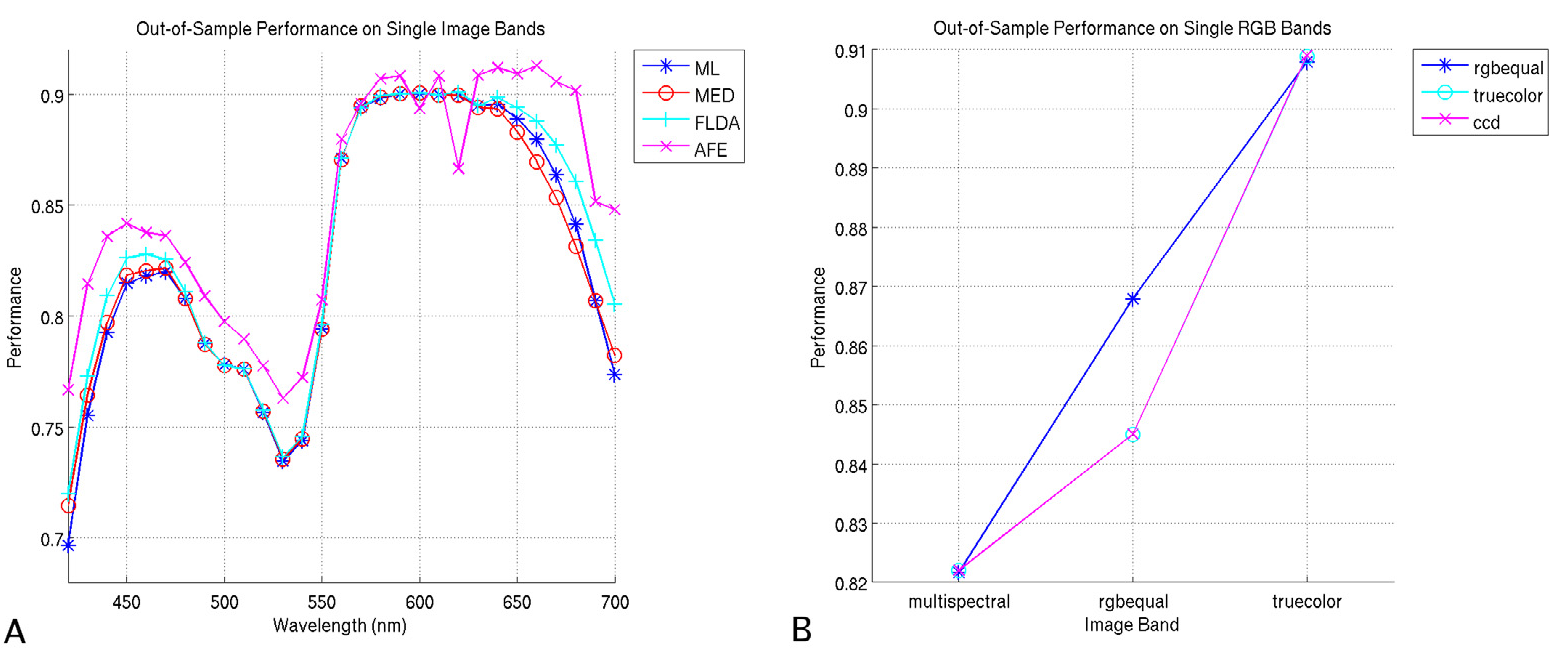
**Performance on single image bands**. (A) Out-of-sample performance scores on single multispectral bands. (B) Out-of-sample performance scores on single RGB bands for the AFE classifier.

To gain a better understanding of the relative contributions of specific image bands, we apply the ML, MED, FLDA, and AFE classifiers to each individual image band for each image type. We exclude the SAM classifier here since it will fail on one-band images, and we exclude the SVM for computational reasons (it would be prohibitively computationally intensive to optimize kernel parameters for each image band). Performance scores for classification using single multispectral bands are shown in Figure 2A. Here we see the best performance scores occurring in the red portion of the spectrum, with poorer performance in the lower green portion and at the extremes of the spectrum.

Similarly, we note that for RGB images, the red channels yield the best performance (Figure 2B); we choose the AFE classifier for presentation here since it consistently yields the highest performance scores, though the other three classifiers display the same trends. While it may seem contradictory that in RGB imagery the green channel outperforms the blue channel when the opposite is true in multispectral imagery, it is important to remember how the multispectral bands are allocated to each of the RGB bands. Consider, for example, the allocation of bands in rgbequal imagery: the bands from 510 nm to 600 nm are averaged to yield the green channel. Referring to Figure 2A we see that these bands have a large variation in performance. Thus, to obtain the green channel, we are averaging multispectral bands, several of which have relatively good performance. A similar situation occurs with the truecolor and ccd imagery, albeit with a weighting applied to each band.

We find the analysis of performance on single image bands satisfactory from an intuitive standpoint. Since the nuclei are stained with the blue-colored Hematoxylin which will block red light, the red portions of the spectrum have the best contrast and perform the best for this nuclear classification task. While green light is also blocked by the Hematoxylin, so also is it blocked by the Eosin, rendering the green portion of the spectrum less informative for the task at hand.

The distinction in performance of red channels between the RGB image types is not large; we do note, however, that the single best performing multispectral band yields a performance increase of 0.57% as compared to the single best RGB band, averaged over all 4 classifiers. This performance increase is consistently in favor of single multispectral image bands, but are not generally statistically significant (refer to Table 2).

**Table 2:** Wilcoxon p-values for performances of the best multispectral band versus the red RGB channel.

**Classifier**	**Band**	**Band**		
		rgbequal R	truecolor R	ccd R
**ML**	multi 590 nm	**0.0316**	0.3086	0.3389
**MED**	multi 600 nm	**0.0218**	0.2452	0.1714
**FLDA**	multi 620 nm	**0.0017**	0.2452	0.3600
**AFE**	multi 660 nm	0.0937	0.4048	0.4653

Wilcoxon paired signed-rank test p-values are presented to 5 significant digits, and bold entries correspond to statistical significance at the p-value of 0.05.

We have shown in this section that performance differences between single multispectral image bands and single RGB image bands are not statistically significant. This would seem to indicate that the individual multispectral image bands are not yielding any more specific spectral information than are the individual RGB image bands for this nuclear classification task.

### Analysis of FLDA coefficients and bands chosen in AFE solutions

**Figure 3:**
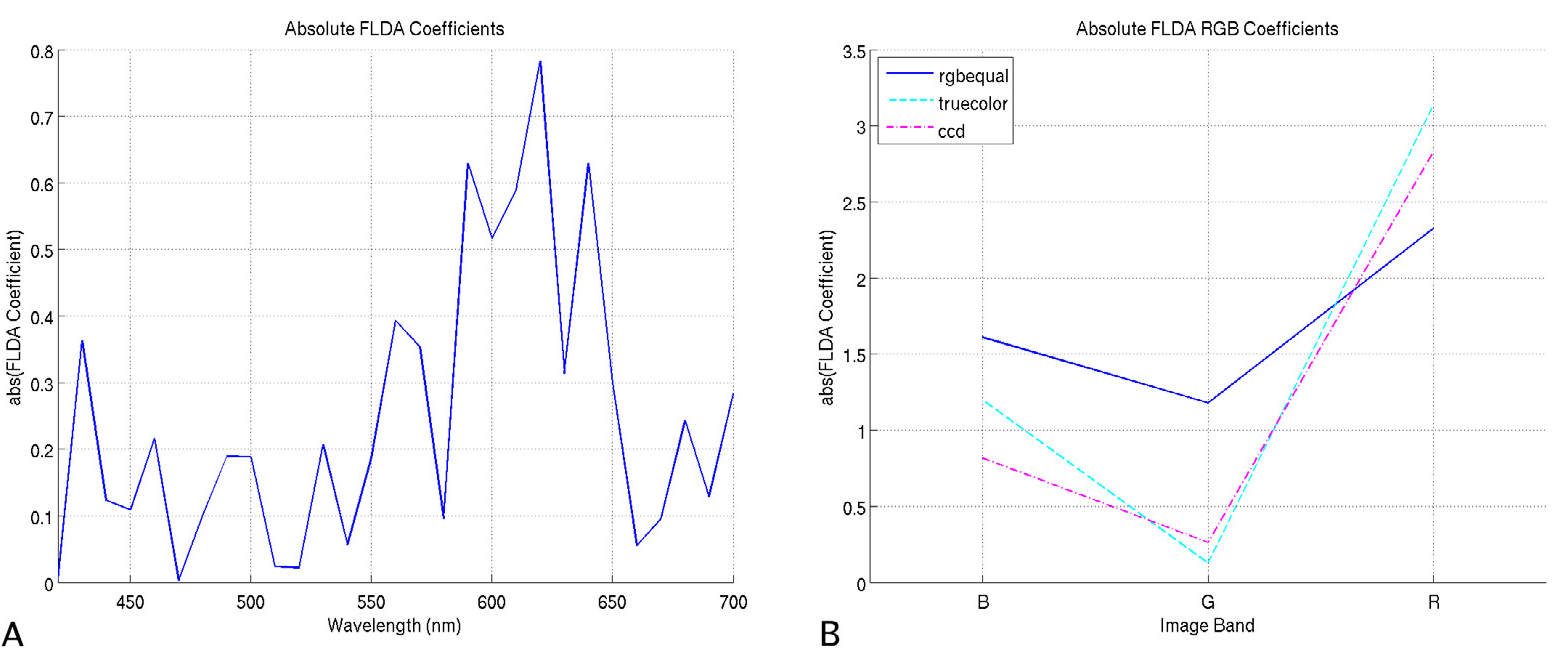
**FLDA coefficients**. (A) FLDA coefficients for multispectral imagery. (B) FLDA coefficients for RGB imagery.

**Figure 4:**
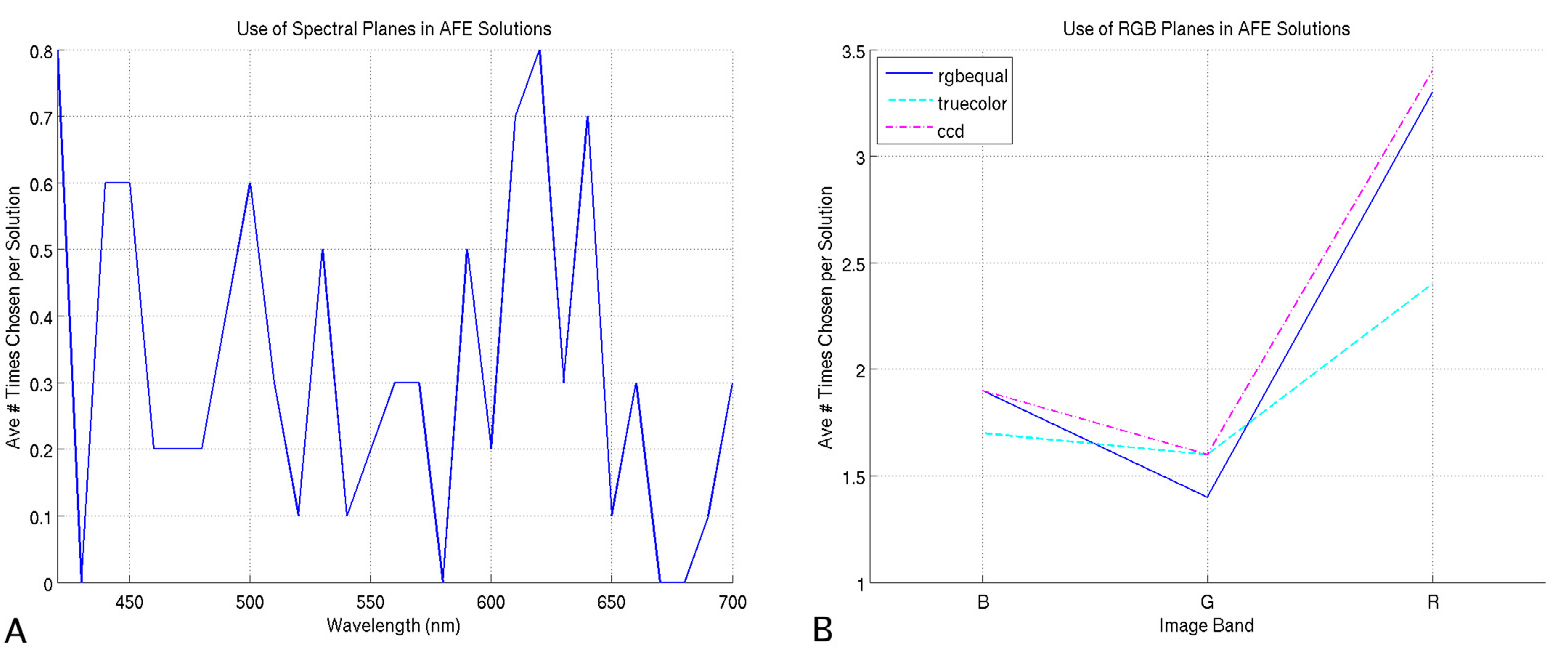
**Spectral bands chosen in AFE solutions, averaged over 10 independent runs**. (A) Multispectral bands chosen in AFE solutions. (B) RGB bands chosen in AFE solutions.

We expect that the single image bands which yield the best performance should also be the bands used most often by the classifiers. A direct examination of this is possible with the FLDA and AFE classifiers. For FLDA, image bands are weighted and summed; the higher the absolute value of the coefficient, the more important the image band. Plots of these coefficients for multispectral and RGB imagery are shown in Figure 3. For the AFE classifier, more important image bands should be chosen more often in solutions; plots of the average number of times an image band is chosen in an AFE solution are shown in Figure 4, where the 10 independent runs have been averaged. Once again, in both the FLDA and AFE classifier, we note a preference for the red portion of the spectrum.

We note also that with RGB imagery, the FLDA classifier weights the red channel the most, followed by the blue, and finally green channels. Similarly, the AFE classifier chooses the red channel most often, followed in turn by blue and green. Comparing the multispectral plots for the AFE and FLDA classifiers, there are striking similarities in the relative use/weighting of bands, particularly in the red portion of the spectrum (i.e., 580–650 nm). The more prevalent use of green and blue bands in the AFE classifier, compared to FLDA, may be due to the classifier's ability to extract local features, making those bands more useful beyond the raw spectral attributes used by the FLDA classifier. Overall, considering the disparate nature of these two classifiers, we find it very interesting that they both display similar preferences for particular image bands.

We use the analysis in this section as a complement to the analysis of performance on single image bands. Specifically, we have shown that image bands that yielded the better performances are also the image bands chosen preferentially in both the FLDA and AFE classifiers. While it may be more qualitatively satisfying if the plots of Figures 3 and 4 would bear more resemblance to those of Figure 2, it is important to remember that these two analyses are very distinct from one another. In the case of Figure 2, we are limiting the classifiers to a single image band, and optimizing the performance, whereas for Figures 3 and 4 we are providing the classifiers with a choice of all available image bands and optimizing performance. As a more intuitive example, for the FLDA classifier, even if a specific image band X performs well when used alone, this same image band X may not yield as much information as, say, the linear combination of bands Y and Z. We have shown, therefore, in this analysis, a classifier preference for image bands which yield better performance when used singly in classification.

### Principal components analysis of image stacks

We use Principal Components Analysis (PCA) as a dimensionality reduction method to see how many "important" bands actually exist within our multispectral image stacks. We choose PCA rather than another dimensionality reduction technique, such as Independent Components Analysis (ICA), since PCA has a well established ranking for the resulting vectors. While there has been at least one ranking method suggested for ICA, the ratio of between-class to within-class variance [18], there is not a universally accepted ranking for ICA vectors. While ICA may yield a better separation of the independent causes in our data (i.e., the two stains), we are interested in the use of a dimensionality reduction technique mainly to help interpret the (lack of) differences in performance we have presented for our multispectral and RGB imagery.

**Figure 5:**
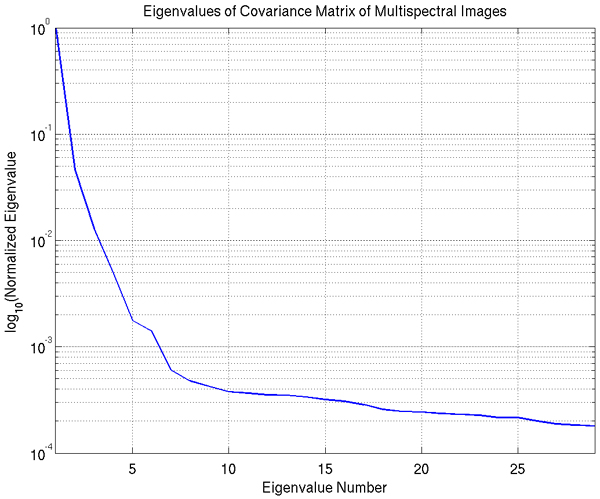
**Logarithmic plot of the eigenvalues of multispectral imagery, from PCA**. Eigenvalues for each image are normalized so that the largest eigenvalue has unit value.

**Figure 6:**
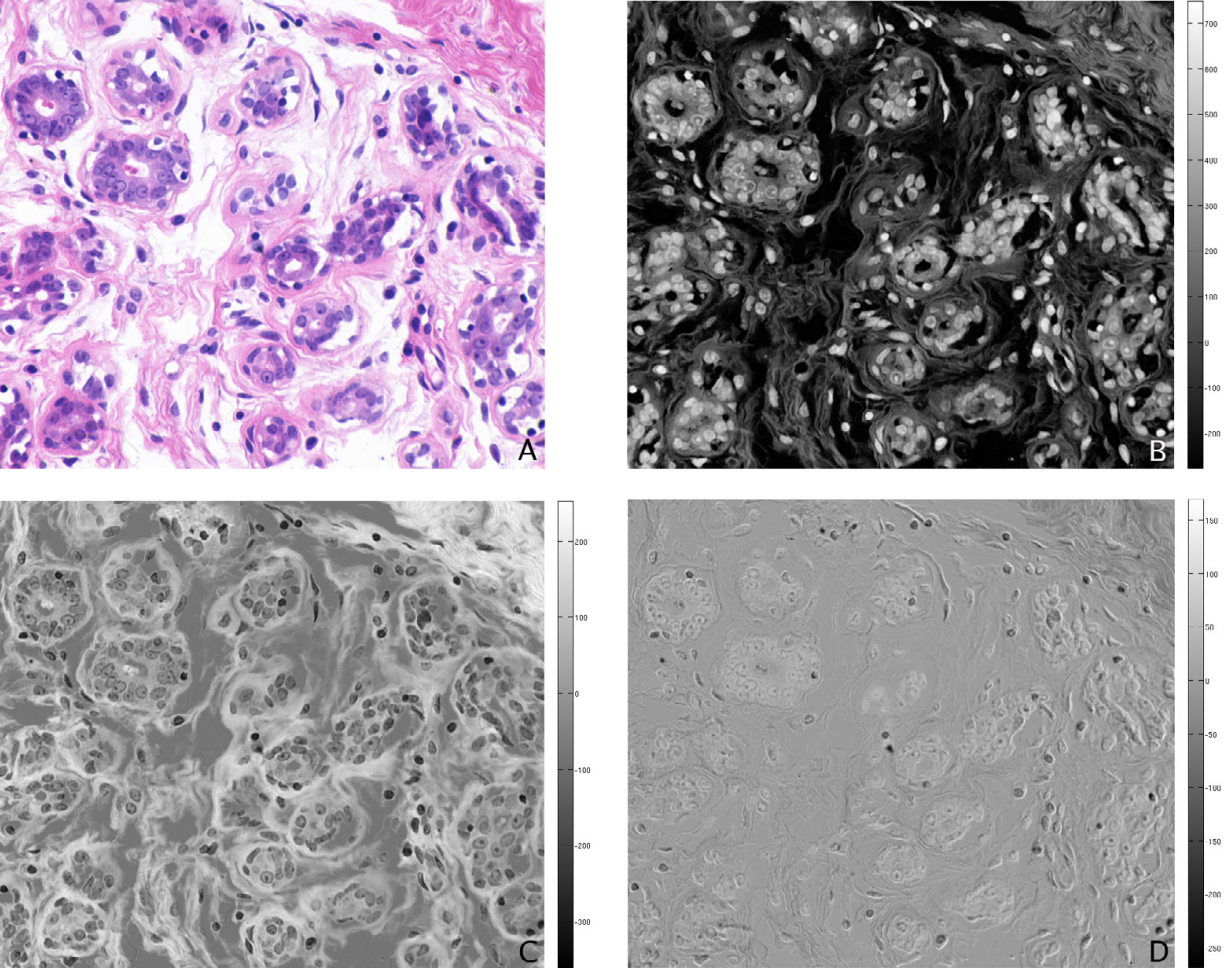
**Projection of example image onto first three eigenvectors**. (A) Projection on first eigenvector. (B) Projection on second eigenvector. (C) Projection on third eigenvector.

As input to the PCA algorithm, we use the (768·896) × 29 matrix where the rows correspond to a single image pixel and the columns are the pixel values for each of the 29 multispectral image bands. We plot the average sorted eigenvalues of the covariance matrix of this input in Figure 5, where for each image we normalize the eigenvalues so that the largest eigenvalue has unit value. We note that there appears to be one dominant eigenvalue, with the second ranked eigenvalue at approximately one-tenth the value of the dominant one; given the two stains in our histopathology imagery, we expected two dominant eigenvalues. We show in Figure 6 the projection of an example image onto the first three eigenvectors. The first projection seems to highlight nuclear regions (i.e., the Hematoxylin), the second projection seems to highlight the connective tissue and cytoplasm (i.e., the Eosin), and the third and subsequent projections do not have any obvious correlation with the tissue stains.

We have thus found that PCA indicates the presence of 2 dominant eigenvalues, when we consider the principle components responsible for 97% of the variation in the data. This indicates the presence of only 2 information-bearing bands in the imagery for this nuclear classification task, providing insight into the approximately equivalent performance of the RGB imagery and multispectral. We have also shown that these 2 informative bands demonstrate a direct relationship to the two image stains. Interestingly, the first component is responsible for 93% of the total variation; this band is generally correlated with Hematoxylin, but is sometimes correlated instead with Eosin. The possibility that other image bands may contain important diagnostic information for further analysis is still an open question [2].

## Conclusion

We have shown a demonstration of performance for different image types and different classifiers in a nuclear classification task. Results seem to indicate only slight performance differences (less than 1%) using multispectral imagery as opposed to our derived RGB imagery; while these performance increases are small, we report them here since they are a direct result from our experiments, and may be statistically significant. These conclusions hold for both classification using all available image bands as well as using single image bands, indicating that the multispectral bands do not contain much more discriminatory spectral information than do the RGB bands for this nuclear classification task. There are, undoubtedly, a number of metrics that could be used in a study such as this, and we may have been able to find a metric for which multispectral would fare better (or worse) than presented here. However, we wanted to use a metric that provides an equal trade-off between two commonly used metrics (detection rate and false alarm rate). We have also shown that the single image bands with the best performance are the image bands chosen more often/weighted more heavily by the AFE and FLDA classifiers. Finally, we have shown through the use of PCA as a dimensionality reduction method, that only 2 image bands are carrying 97% of the variation in our image data, and appear to be correlated with the two image stains. This result provides some insight into the roughly equivalent performance of RGB imagery to multispectral. While the results presented here are intriguing, they are by no means complete, since we are considering only a single pixel-level classification task. Future work will continue to compare multispectral with RGB imagery for further classification tasks, as well as other image analysis tasks, including object-level analysis. In particular, we are currently researching methods to segment (i.e., delineate) individual nuclei using the results of these pixel-level classifications.

## Methods

### Sample preparation and image acquisition

**Figure 7:**
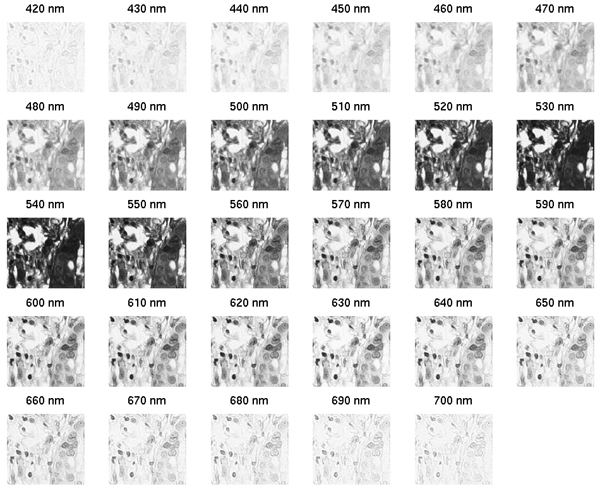
**Example multispectral stack**. Only a portion of the 768 × 896 image is shown.

Our dataset contains 58 H&E stained histopathology images of breast tissue from the Yale Tissue Microarray Facility [19]. The data was captured from 5 microarrays (ytma10, 12, 49, and 55), with (6, 6, 34, and 6) images captured per array, respectively; in total we have 26 malignant images, and 32 benign (including 6 normal from ytma55). Our 58 images are not microarray images in the general sense since we are dealing with single histopathology images as might be obtained from standard clinical biopsy specimens. The multispectral images have 29 bands, spaced 10 nm apart, ranging within the visible spectrum from 420 to 700 nm, acquired using the VariSpec™ (CRi, Woburn, MA) liquid crystal tunable filter and a typical clinical pathology microscope setup with a 40× objective (400× total magnification). Each band is represented in an image stack as an 8 bit, 768 × 896 grayscale image; an example is shown in Figure 7. It should be noted that each image band has been corrected for illumination differences via a flat-fielding operation; this is part of the acquisition software included with the VariSpec™.

### Derivation of RGB imagery

One could foresee many methods for the derivation of RGB imagery from multispectral. We use here:

1. **rgbequal**: created by (approximately) equally allocating the 29 bands to R, G, and B, similar to the approach in [7], reflecting a rough approximation of the three spectral ranges associated with the three colors red, green, and blue, albeit with some ambiguity in allocation of intermediate colors (e.g., yellow).

2. **truecolor**: created by converting the illumination wavelength for each band into the constituent RGB values as perceived by humans, then averaging the contribution to R, G, and B for each band. This method utilizes the MatlabCentral [20] function *spectrumRGB*.

3. **ccd**: a modification of truecolor imagery to better match the spectral response of common 3-CCD color cameras used in microscopy setups for biomedical research. This method also utilizes the *spectrumRGB *function.

**Figure 8:**
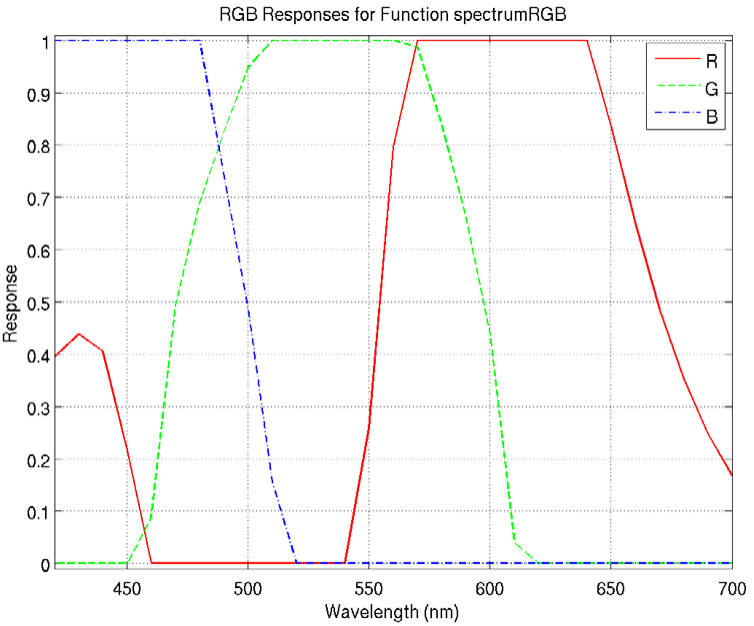
**RGB responses for the function *spectrumRGB***. Note the second lobe of the red response in the smaller wavelengths. This is due to the perception of such wavelengths as violet, represented in RGB as a combination of red and blue. The ccd image representation removes the contribution of this second lobe.

**Figure 9:**
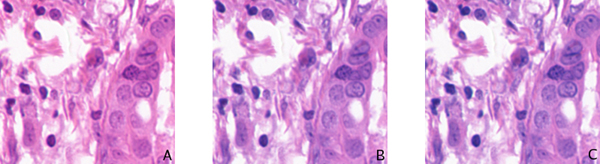
**RGB representations of the example multispectral stack of Figure 1**. The same portion of the 768 × 896 image is shown here. (A) Bands allocated equally and averaged, "rgbequal" (B) Bands allocated with MatlabCentral function *spectrumRGB*, "truecolor." (C) Bands allocated to approximate spectral responses of common 3-CCD color cameras, "ccd."

It should be noted that the ccd and truecolor representations differ only in the red band. The RGB responses of the function *spectrumRGB *function are shown in Figure 8 and examples of each of these three types of RGB images are shown in Figure 9.

### Classifiers

We describe here the six pixel-level classifiers used in this study. We choose these classifiers based on their established performance and use for multispectral data, sparsity of parameters to optimize, computational efficiency, and the use of (primarily) spectral information. The use of primarily spectral information is important in these analyses since the basic hypothesis in question deals with the spectral information content of our imagery. The exceptions to these characteristics are noted in the classifier descriptions to follow.

• **Maximum Likelihood (ML) **[21]: Maximizes the likelihood of a pixel belonging to a certain class. That is, a pixel is assigned the label of the class that it is most likely to be a member of. Likelihood is defined probabilistically, using the estimated joint probability density or mass function. We assume a Gaussian density model, and estimate the mean and covariance matrix for each class. These assumptions result in a quadratic discrimination boundary.

• **Minimum Euclidean Distance (MED)**: Minimizes the Euclidean distance between an observation and the class means.

• **Spectral Angle Mapper (SAM)**: Minimizes the angle between an observation and the class means.

• **Fisher Linear Discriminant Analysis (FLDA)**: Projects the multi-dimensional data to one dimension, maximizes a function representing the difference between the projected class means, and normalizes by the within-class scatter along a direction perpendicular to the decision hyperplane [22]. This is also equivalent to a Maximum Likelihood formulation assuming equal covariance matrices for each class, resulting in a linear discrimination boundary.

• An **Automated Feature Extraction (AFE) **tool called **GENIE**: GENIE is based on evolutionary computation, and is designed to explore the entire feature space of multispectral data, and evolve a solution best fit for the classification task. More practically speaking, GENIE selects an initial set of algorithms consisting of randomly selected operators and randomly selected data planes as input. Throughout the evolution process, only appropriate algorithms with appropriate data input will survive. GENIE has the ability to use information from both the spectral and spatial domain, which renders it unique among the six classifiers. For more information, see Reference [23].

• **Support Vector Machine (SVM)**: Constructs a linear hyperplane that maximizes the margin between classes. In the case of nonlinear SVMs, the data is first mapped to a higher dimensional space where a linear hyperplane is computed to separate the classes, using a kernel function which defines the inner product operation in the higher dimensional space [24]. We have implemented an SVM using SVM^*light *^[25], with a linear kernel (**LSVM**) using all training data as input, and a quadratic kernel (**NLSVM**) using a randomly selected 10% of our training data as input (to speed the training process to a reasonable time). For this classifier, the kernel parameters must be explicitly optimized for the training data; this is the only classifier used in this study which requires optimization of parameters.

Before discussing our performance metric and results, we would like to briefly discuss how these pixel-level nuclear classifications will be used. We are currently working towards a hierarchical image analysis system, where we will alternate classification and segmentation of the imagery in an interactive system eliciting user feedback. Current active research involves nuclear segmentation, i.e., the proper delineation of all nuclei contained in the image. As such, it is necessary to achieve an accurate classification of all nuclei pixels if we are to use shape and other appropriate metrics to their best advantage in the nuclear segmentation process.

Humans inherently incorporate higher-level information in their analysis of imagery; since we are considering the nuclear classification performance based on primarily spectral information, it is difficult, if not impossible, to specify the expected level of performance for a human expert. The issues of human performance in diagnosis, particularly the inter- and intra-observer variability (see [26,27] and the references therein) will be an important consideration in our future work and is indeed a strong motivation for a computerized quantitative analysis.

### Performance metric

We choose a general metric of classification performance that equally penalizes both types of classification errors: 1) true (nuclei) pixels incorrectly labeled as false (non-nuclei) and 2) false pixels incorrectly labeled as true. In particular, the performance metric is defined as

*P *= 0.5(*R*_*d *_+ (1 - *R*_*f*_)),

where *R*_*d *_is the fraction of true pixels classified correctly (detection rate), *R*_*f *_is the fraction of false pixels classified incorrectly (false alarm rate), and the factor of 0.5 scales the metric to the range [0, 1]. Note that a perfect segmentation will yield a performance score of 1 (100%), while a score of 0.5 (50%) can be obtained by a trivial solution of all pixels labeled as a single class (true or false). This metric is an equal tradeoff between detection rate and false alarm rate.

As a compromise between the necessity of comprehensive ground truth for proper quantification of classification accuracy, and the tedious and time-consuming aspect of human delineation of such ground truth, we have marked a 200 × 200 pixel window in each of our 58 histology images. This window is used to determine classification performance for each image.

## Abbreviations

ML – Maximum Likelihood

MED – Minimum Euclidean Distance

SAM – Spectral Angle Mapper

FLDA – Fisher Linear Discriminant Analysis

AFE – Automated Feature Extraction

SVM – Support Vector Machine

LSVM – Linear Support Vector Machine

NLSVM – Non-Linear Support Vector Machine

PCA – Principal Components Analysis

ICA – Independent Components Analysis

## Competing interests

The authors declare that they have no competing interests.

## Authors' contributions

LEB performed analysis of the image data, including development of appropriate quantitative tests, necessary coding, and compilation and interpretation of the results, as well as drafting and revising the manuscript. ZB conducted the SVM classifications and provided technical expertise related to SVMs. NRH helped develop the test process, participated in the the interpretation of the results, and assisted with the preparation of the manuscript. BSM participated in the interpretation of results, suggestions for further analysis, and provided technical feedback for the final drafting of the manuscript. DLR provided pathology expertise, imagery, and technical feedback for the manuscript. All authors read and approved the final manuscript.
